# The Relation between Post-Endoscopic Retrograde Cholangiopancreatography Pancreatitis and Different Cannulation Techniques: The Experience of a High-Volume Center from North-Eastern Romania

**DOI:** 10.3390/life13061410

**Published:** 2023-06-19

**Authors:** Stefan Chiriac, Catalin Victor Sfarti, Carol Stanciu, Camelia Cojocariu, Sebastian Zenovia, Robert Nastasa, Anca Trifan

**Affiliations:** 1 Department of Gastroenterology, “Grigore T. Popa” University of Medicine and Pharmacy, 700115 Iasi, Romania; stefannchiriac@yahoo.com (S.C.); sebastianzenovia20@gmail.com (S.Z.); robertnastasa948@gmail.com (R.N.); ancatrifan@yahoo.com (A.T.); 2 Institute of Gastroenterology and Hepatology, “St. Spiridon” Emergency Hospital, 700259 Iasi, Romania; stanciucarol@yahoo.com

**Keywords:** ERCP, post-ERCP pancreatitis, cannulation techniques

## Abstract

Background: Despite numerous advances that have aimed to increase the safety of endoscopic retrograde cholangiopancreatography (ERCP), post-ERCP pancreatitis (PEP) still remains a major issue. We aimed to assess the rate of PEP as well as the relation to the cannulation techniques in our unit, a high-volume center in north-eastern Romania. Methods: ERCPs performed in our unit from March to August 2022 were retrospectively included. Data concerning demographic information, presence of difficult cannulation, the technique used for cannulation, as well as immediate complications, were gathered from the electronic database. Results: 233 ERCPs were included. PEP was diagnosed in 23 (9.9%) of cases. Precut sphincterotomy (PS), transpancreatic sphincterotomy (TPBS), and a combination of TPBS and PS were performed in 6.4%, 10.3%, and 1.7% of cases, respectively, while an Erlangen precut papillotomy was performed in one case. Both in patients with PS and TPBS the rate of PEP was 20%. When the two techniques were associated, the rate of PEP was 25%. TPBS and PS represented risk factors for PEP (OR 1.211 for a CI of 0.946–1.551, *p* = 0.041, and OR 1.124 for a CI of 0.928–1.361, *p* = 0.088, respectively). No PEP-associated deaths were found. Conclusions: Both PS and TPBS presented a similar risk of PEP.

## 1. Introduction

Post-endoscopic retrograde cholangiopancreatography (ERCP) pancreatitis (PEP) is the most frequent complication of ERCP, accounting for high morbidity, prolonged hospitalization, and great financial costs [1,2]. The pathophysiology of PEP is not entirely elucidated, but it comprises several factors that ultimately lead to the intracellular activation of the pancreatic enzymes, followed by autodigestion and the involvement of a cascade of cytokines, responsible for local as well as systemic inflammation [3]. Trauma to the papilla caused by a guidewire and/or sphincterotome during cannulation plays a central role, as it can produce papillary edema. Consequently, a transient obstruction of the pancreatic juice outflow may lead to the increase in ductal pressure and to the development of PEP [4]. Unintentional contrast injection into the pancreatic duct, which can occur especially in the setting of difficult cannulation, may also induce PEP via hydrostatic damage [1,5].

Cannulation of the papilla represents the first step in ERCP, but easy access to the common bile duct (CBD) is not always possible. Difficult cannulation has been defined by the European Society of Gastrointestinal Endoscopy (ESGE) as more than five contacts with the papilla or cannulation attempts over 5 min or more than one unintended pancreatic duct cannulation, and these have been associated with a high risk of PEP [1]. If easy cannulation of the papilla is not possible, different cannulation techniques, such as needle-knife precut sphincterotomy (PS), the double-guidewire technique, or transpancreatic sphincterotomy (TPBS), can be used [1,6]. The first occurrences of biliary sphincterotomy were documented by researchers in Erlangen, Germany, and Kawai [7] in Japan, who were independently working on therapeutic uses of ERCP. The “Demling-Classen” probe made it possible to successfully perform a sphincterotomy and administer contrast dye while the catheter was still in place. However, each of these techniques has been associated with an additional risk of PEP development. Controversy still exists regarding the optimal choice of cannulation, but also regarding the ideal moment when it should be used [8]. Endoscopists can employ a rendezvous approach to accomplish selective biliary cannulation (SBC) when precut procedures fail or if the anatomy of the papilla prevents the use of precut techniques. Any method of biliary or pancreatic ductal cannulation that involves inserting a wire anterogradely through the papilla and into the duodenum, followed by SBC either over the wire itself or in parallel to the wire, is known as a rendezvous technique. A well-known salvage approach called endoscopic ultrasound (EUS)-guided rendezvous (EUS-RV) involves directly puncturing the biliary ducts from the gastric or duodenal lumen under real-time EUS guidance. The wire is then advanced anterogradely through the needle, into the duct, and out the papilla. The sphincterotome can then be directed over the guidewire or parallel to the wire to accomplish SBC [9]. A significantly higher success rate (98.3% versus 90.3%, *p* = 0.03) was observed in the EUS-RV group in a retrospective study of 58 patients who underwent EUS-RV and 144 who received the Erlangen precut cannulation technique [10]. There were no differences in the complication rates (3.4% versus 6.9%, *p* = 0.27) or episodes of pancreatitis in the EUS-RV group. The increased technique time, equipment, and training needed to perform EUS-RV, as several authors have noted, may represent a limitation in the general implementation of this procedure [11,12]. Most typically, strictures, masses, or edema can prevent a guidewire from passing anterogradely through the papilla, leading to EUS-RV failure. Additionally, the procedure raises the possibility of perforation and biliary peritonitis. When an EUS-RV fails, salvage procedures include direct ampulla puncture under EUS supervision, rendezvous re-attempts after EUS-cholangiography, and hybrid rendezvous, which entails employing a dilator to widen the needle tract while managing the wire [13,14]. There are several other cannulation techniques in which either a guidewire or a stent is placed in the pancreatic duct, aiming to facilitate the achievement of SBC. A guidewire in the main pancreatic duct (MPD) lowers the possibility of unintentional cannulation of the MPD by helping to straighten the intramural section of the bile duct and guiding the sphincterotome or other catheter into the bile duct. The double-guidewire technique (DGT) is the name given when the pancreatic guidewire approach is used in conjunction with wire-guide cannulation. There was no difference in the success rate of cannulation or the rate of PEP between a retrospective analysis with 363 participants and a prospective multicenter RCT with 274 patients comparing the pancreatic guidewire technique to early DGT [15,16]. The use of DGT, however, increased the incidence of PEP when compared to other approaches, such as standard wire-guided cannulation, MPD, and early precut (RR = 1.98, 95% CI). This was stated by Tse et al., in a recent meta-analysis of 7 RCTs involving 577 patients [17]. As all retrograde cannulation techniques have been associated to some degree with PEP, over time, a high number of drugs, such as antibiotics, heparins, corticosteroids, nifedipine, octreotide, and somatostatin derivatives, trinitrin, lidocaine spray, gabexate, secretin, topical epinephrine, and cytokine inhibitors have been tested experimentally and in clinical trials for potential efficacy in the prevention of this complication. However, only nonsteroidal anti-inflammatory drugs (NSAIDs) gathered adequate evidence of their effectiveness [3]. In all patients who have no contraindications, the ESGE proposes routine rectal administration of 100 mg of indomethacin or diclofenac either immediately before or after ERCP. If NSAIDs are contraindicated, it is optional to deliver sublingual glyceryl trinitrate in high-risk situations [5]. Moreover, current clinical practice guidelines, based on observational studies, recommend aggressive hydration to prevent PEP, as it has been shown to present a protective role [18,19,20]. Since its inception 50 years ago, ERCP’s therapeutic potential has significantly increased. One thing that has remained unchanged is that successful ERCP depends on having the right training, applying rigor in performing the procedure in patients with a clear indication for ERCP, positioning the duodenoscope before the papilla in the right position before attempting cannulation, and choosing the adequate initial cannulation techniques. When employing the traditional methods of contrast-assisted or wire-guide cannulation, even in the case of the ideal patient, SBC was not achieved in up to 20% of cases; these cases were classified as difficult cannulations and required additional techniques [9].

We aimed to perform an analysis of the risk of PEP regarding the use of different cannulation techniques in our center.

## 2. Materials and Methods

### 2.1. Study Design

The study, a retrospective service evaluation, included ERCPs performed in the “St. Spiridon” Emergency Hospital, Institute of Gastroenterology and Hepatology, Iasi, Romania, a tertiary high-volume center [21]. ERCPs performed in our unit from March to August 2022 were retrospectively included. Data concerning demographic information, difficult cannulation, the technique used for cannulation, as well as immediate complications were obtained from the electronic database in a confidential manner. The indication, endoscopic diagnosis, procedures performed, and success rates were also noted. Incomplete records were excluded from the study.

### 2.2. Definitions and Outcomes

The morphology of the papilla of Vater was noted according to the classification proposed by Haraldsson et al., as follows: Type 1, regular, unremarkable aspect of the papilla; Type 2, a flat papilla less than 3 mm diameter; Type 3, a protruding, pendulous papilla; Type 4, ridged or creased papilla [22].

Difficult cannulation was defined as more than five contacts with the papilla or cannulation attempts over 5 min or more than one unintended pancreatic duct cannulation [1]. The cannulation was considered successful if deep biliary cannulation with the guidewire or sphincterotome was obtained.

PEP was defined as new or worsened abdominal pain combined with >3 times the normal value of amylase or lipase at more than 24 h after ERCP and the requirement of admission or prolongation of a planned admission [1].

The main aim of the study was the analysis of the risk of developing PEP in regard to the type of cannulation. Secondary outcomes included the rate of PEP, as well as the presence of other significant post-ERCP complications, namely bleeding and perforation.

### 2.3. PEP Prevention and Procedure Description

The procedures were performed according to ESGE recommendations, with patients under general anesthesia, supervised by the intensive care unit (ICU) team. Immediately, before the procedure, all patients without contraindications received nonsteroidal anti-inflammatory drugs (NSAIDs). In patients with contraindications to NSAIDs as well as in those with difficult cannulation but with low risk of fluid overload, aggressive hydration with Ringer solution was started during the procedure and continued as recommended by the intensivist. The procedures were performed by endoscopists with high expertise. 

**Figure 1:**
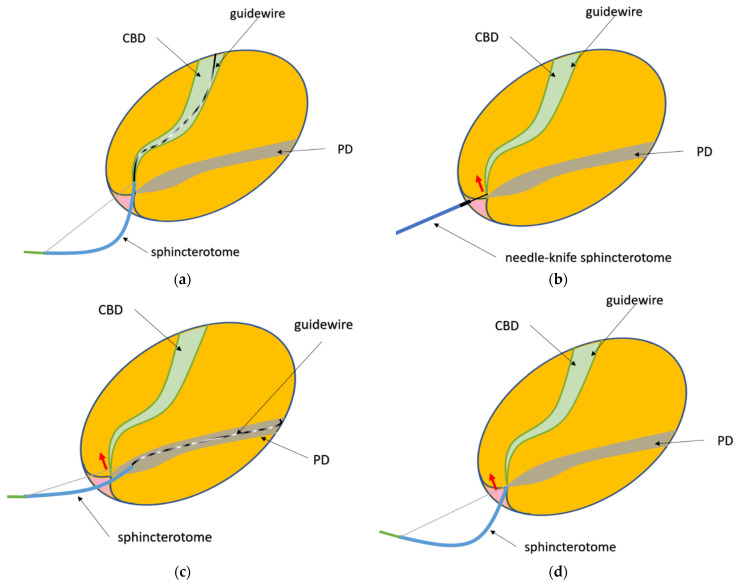
Illustrations of the techniques used to access the common bile duct. (**a**) Standard cannulation technique; (**b**) precut sphincterotomy; (**c**) transpancreatic sphincterotomy; (**d**) Erlangen precut papillotomy.

The duodenoscope was inserted into the second part of the duodenum, at the level of the papilla under direct visualization. The ERCPs were mostly carried out with the duodenoscope in the “short” position, but the “long” position was occasionally used, at the discretion of the endoscopist. The major duodenal papilla was initially cannulated with the aid of a standard pull-type sphincterotome pre-loaded with a hydrophilic guidewire. The wire-guided technique was used in all cases. In the case of difficult cannulation but with easy access to the PD, TPBS was performed; subsequently, further CBD cannulation with the guidewire-loaded sphincterotome was attempted. If access to the PD with the guidewire was not possible or if TPBS was not followed by deep cannulation of the CBD, PS was carried out. Erlangen precut papillotomy was performed in one case. Prophylactic pancreatic stents were placed at the discretion of the endoscopist. The techniques used are illustrated in Figure 1.

### 2.4. Statistical Analysis

The statistical analysis was performed using IBM Statistical Package for Social Sciences (SPSS) version 22.0. The continuous variables were assessed using the Kolmogorov–Smirnoff test and the distribution was nonparametric. Thus, the variables were expressed as median (interquartile range, IQR). Categorical variables were expressed as frequency as well as percentage; subsequently, the Chi-square or Fischer’s test were used for their analysis. Statistical significance was considered for a *p* value of less than 0.05. 

## 3. Results

### 3.1. Patients

A total of 243 ERCPs were performed on distinct patients during the six-month period. After the exclusion of incomplete records, 233 ERCPs were included in the study. The median age of the subjects was 68 years old (57–76), and there were 119 (51.1%) women. The median body mass index (BMI) was 23 kg/m^2^ (21–24). Periampullary diverticula (PAD) were present in 27 (11.5%) of the cases. Most patients presented unremarkable, Type 1 papillary anatomy (64.8%). Concerning the indication for ERCP, the majority of procedures were performed for benign conditions (75.1%), among which the main indication was CBD stones (64.4%). The general characteristics of the patients are presented in Table 1.

### 3.2. ERCP Characteristics

General cannulation success was obtained in over 90% of cases. Most frequently, deep cannulation of the CBD was achieved using the wire-guided standard technique (81.1%). In 30% of cases, CBD cannulation was deemed difficult; therefore, advanced techniques were performed. Most frequently, when access to the CBD was difficult but there was unintentional PD cannulation, TPBS was performed (10.3% of cases). In 6.4% of cases, neither the CBD nor the PD could be easily accessed; thus, PS was carried out. In 1.7% of cases, after an initial TPBS, wire-guided attempts to cannulate the CBD were not successful. Therefore, TPBS was followed by PS during the same procedure. Pancreatic stents were placed in 12% of patients, as indicated by the endoscopist, in the case of repeated pancreatic duct cannulation as well as in the case of TPBS.

The cannulation of CBD was deemed difficult in 30% of the cases. All of the patients that had Erlangen precut papillotomy, PS, TPBS, and the association of these techniques performed as well as 26 of the cases from the standard wire-guided cannulation group presented initial difficult cannulation.

The general rate of PEP was 9.9%. One patient developed severe PEP and required admission to the intensive care unit. There were no PEP-associated deaths. Concerning other significant adverse effects, the most frequent one was early bleeding, which occurred in 1.7% of patients and was treated by submucosal dilute injection of epinephrine followed by thermal coagulation in cases of failure. One patient presented periampullary perforation, which was managed conservatively with a good outcome. The general characteristics of the ERCPs are presented in Table 2.

### 3.3. Analysis of the Risk Factors for PEP and Other Major Post-ERCP Complications

In our analysis, several patient-related as well as procedure-related factors were associated with PEP. Concerning patient-related factors, young age, female gender, and non-standard major papilla morphology increased the rate of PEP, as shown in Table 3. However, a statistically significant risk was found only in the case of high BMI.

The analysis of procedure-related complications showed that 7.4% of the patients that underwent standard wire-guided cannulation developed PEP. When analyzing only the 26 cases of difficult cannulation from the standard wire-guided cannulation group, we found that PEP was diagnosed in 3 (11.5%) of the cases from this subgroup. Furthermore, 20% and 20.8% of the patients that underwent PS or TPBS, respectively, developed PEP. One of the four patients (25%) that had the two techniques performed during the same procedure also developed PEP. Although there was one severe case of PEP, there were no PEP-related deaths. Regarding the risk of bleeding, four patients that underwent wire-guided standard cannulation presented intraprocedural bleeding. PS was followed by bleeding in one patient, while TPBS was associated with bleeding in one patient. One patient that had the association of the two procedures developed bleeding. The only case where an Erlangen precut papillotomy was performed did not develop bleeding. In all of the cases, the bleeding was controlled endoscopically. There was only one case of perforation, and that developed secondarily to PS and TPBS (Table 4).

Univariate risk analysis identified BMI > 24 and TPBS as risk factors for PEP, with an odds ratio (OR) of 2.578 for a 95% confidence interval (CI) between 1.017 and 6.534 and OR 1.211 for a CI of 0.946–1.551, *p* = 0.041, respectively. PS was associated with an OR of 1.124 for a CI of 0.928–1.361, *p* = 0.088, for PEP. Standard wire-guided cannulation was protective for the risk of PEP, OR 0.311, for a 95% CI between 0.125 and 0.775.

## 4. Discussion

Surgical advancements, such as laparoscopic cholecystectomy, but also developments in the field of endoscopy, specifically EUS, and radiologic advancements, namely magnetic resonance cholangiopancreatography, have had a significant impact on the evolution of ERCP, as they have improved pre-procedural diagnostic, thus, establishing correct indications and allowing for ERCP to be performed in appropriate settings. The list of current ERCP indications is extensive and includes, but is not limited to, biliary obstruction due to stones, cancer, infection, type 1 and 2 sphincter of Oddi dysfunction (SOD), palliative and therapeutic ductal stenting, and many more. ERCP has always been an intrusive operation, regardless of the indication; thus, successful completion of this procedure safely calls for extensive expertise and skill [9].

This study aimed to assess the risk of PEP associated with different canulation techniques. We found the general rate of PEP in our institution to be 9.9%, which was within the previously reported range [1,2,3,4,23,24,25].

### 4.1. Patient-Related Risk Factors

There are numerous patient-related risk factors for PEP, such as previous history of PEP or pancreatitis caused by other factors, female gender, younger age, presence of SOD, obesity, and comorbidities, such as congestive heart failure or end-stage renal disease [23]. A multivariate analysis of PEP risk factors revealed their independence; they are also known to work synergistically to raise the rate of PEP, suggesting that they may have an additional effect. Using data prospectively gathered from roughly 2000 ERCPs, Freeman et al., calculated the adjusted OR for various combinations of risk factors. The authors discovered that female patients with a normal serum bilirubin level, SOD, and challenging biliary cannulation had the highest risk of PEP, accounting for 42% of cases [24]. Similarly, a retrospective cohort research by Cheng et al., which examined 1115 patients, showed that age under 60, a history of post-ERCP pancreatitis, and SOD were all risk factors for PEP [25]. In our analysis, we found that a higher percentage of patients under 60 years of age as well as of women developed PEP; however, there was no statistical significance.

Because not all potential risk factors have been examined, the list of identified risk factors is not all-inclusive. Based on limited prospective trials, it was reported that the underlying existence of cirrhosis, primary sclerosing cholangitis (PSC), chronic (autoimmune) hepatitis, Crohn’s disease, and obesity were independent predictors of post-ERCP complications, including PEP [26,27]. Pre-ERCP blood urine nitrogen (BUN) and hematocrit (HCT) levels have been proven to have some value as potential predictors of PEP. A higher incidence of PEP was found to be linked to higher pre-procedure BUN and HCT levels [28]. Smoking, past drinking, and diabetes were all independent risk factors, according to another case-control research including 6505 participants conducted by DiMagno et al., in 2013 [29]. Furthermore, according to a study by Freeman et al., from more than 20 years ago, the likelihood of overall complications, including PEP, was significantly increased when at least one of the independent risk variables (SOD, cirrhosis, difficult bile duct cannulation, PS, or a combination of percutaneous and endoscopic procedures) was present. As a result, overnight stays for post-ERCP patients who displayed one of the listed risk factors have been justified [30]. Nevertheless, it has been shown that chronic pancreatitis protected against PEP, probably as a result of pancreatic atrophy and diminished enzymatic exocrine activity [24]. Pancreas divisum was not found to be a risk factor in and of itself for PEP; however, sphincterotomy of the papilla minor and dorsal duct manipulation enhanced the incidence of PEP [31]. A recent study conducted in the United States of America found that alcohol and cocaine use, chronic kidney disease, and heart failure, as well as obesity, were patient-related risk factors for developing PEP [32]. Similarly, in our analysis, we found that a higher BMI was associated with an increased risk of PEP.

### 4.2. Procedure-Related Risk Factors

Trauma to the papilla is the main physiopathological driver for the development of PEP. This can induce Oddi sphincter spasm, papillary edema, obstruction of the pancreatic juice flow, and a cascade of events that eventually leads to pancreatic injury [3]. Repeated attempts to cannulate the papilla have been shown to be associated with a higher risk of PEP; moreover, each attempt further increases this risk [33]. Thus, measures should be taken in order to avoid this situation. Endoscopists have a range of early precut or rendezvous procedures at their disposal when the anatomy of the papilla is unfavorable or difficult cannulation is predicted. The choice of technique depends on the endoscopist’s experience, the condition being treated, and the anatomy of the patient. When deciding which technique to use during a difficult cannulation, these factors should all be taken into account [9].

In the case of difficult cannulation, current recommendations state that both PS and TPBS can be performed in order to facilitate access to the common bile duct [6]. PS is an established technique that involves using a needle-knife sphincterotome. A cut is performed, either freehand or over a previously placed pancreatic stent, starting from the papillary orifice in a stepwise fashion, towards the direction of the bile duct. Alternatively, a fistulotomy can be carried out, when an incision is performed at the roof of the papilla in an attempt to intercept the CBD [6]. TPBS is a cannulation technique that involves inserting the sphincterotome over a guidewire into the PD and making an incision towards the CBD in an attempt to intercept the bile duct by cutting the septum between the PD and the CBD [34]. However, these procedures carry a supplementary risk of PEP. The risk of developing PEP is deemed similar between TPBS and PS, with an odds ratio between 2.11–3.1 for PS and 1.23–3.07 according to ESGE guidelines [1]. However, there are reports of an increased incidence of PEP when TPBS is performed rather than PS (29.8% versus 12.7% of cases, respectively) [24]. A more recent retrospective 5-year analysis of 1082 cases reported a low rate of 2.8% of cases of PEP following TPBS, with a RR of 0.015, 95%CI between 0.23–5.05 for a *p* < 0.001, compared to a RR of 3.104, 95%CI between 1.03–9.36 for a *p* = 0.04 in the case of PS [34].

As these techniques usually follow repeated cannulation attempts, the risk of PEP could actually be related to the prolonged instrumentation of the papilla rather than to the technique used. Supporting this theory are a series of studies that analyzed the impact of early PS on the rate of PEP; the findings are consistent with a lower risk of developing PEP following early PS in contrast with repeated attempts of cannulation followed by late PS [35]. Several studies indicated a considerable reduction in the risk of PEP in patients having early PS versus usual procedures. Prior to randomizing to PS versus persistence with standard cannulation techniques, these studies used a heterogeneous definition for difficult biliary cannulation, specifying a duration of attempted cannulation of >5 to 12 min or >2 to 4 unintentional pancreatic duct cannulations [36,37]. We found that in the setting of difficult cannulation, as defined by ESGE criteria, the rates of PEP were higher in patients that required PS or TPBS. The highest rates of PEP were found when both PS and TPBS were associated. These findings should, however, be interpreted with caution, as the total procedure time and the length of cannulation could not be analyzed because of lack of documentation. Thus, the higher rates of PEP could be related to prolonged instrumentation, especially in complex cases that required both PS and TPBS.

Another option in the setting of difficult cannulation is the use of an Erlangen precut papillotomy. This procedure traditionally involves the use of a dedicated sphincterotome. The instrument is insinuated into the papillary orifice and a cut towards the biliary orifice is performed without the prior use of guidewire or contrast medium injection. Thus, the unroofing of the papilla provides access to the CBD with reduced risk of PEP [8,9]. In our study, an Erlangen precut papillotomy was performed in only one case, and no complication was noted.

Endoscopic papillary large-balloon dilation (EPLBD) of an intact biliary sphincter for the extraction of large choledocholithiasis was found to have considerably greater rates of PEP than biliary sphincterotomy in 15 randomized clinical trials involving 1768 patients [38]. According to a prospective randomized study, a 1 min EPLBD with an intact biliary sphincter was linked with a greater incidence of PEP than a 5 min dilatation (15.1% versus 4.8%; *p* = 0.038) [39]. A further meta-analysis published by Liao et al., in 2012 found that EPLBDs lasting less than one minute had greater rates of pancreatitis than EPLBDs lasting more than one minute [40]. On the other hand, balloon dilation following biliary sphincterotomy, or adjunct balloon sphincteroplasty, did not raise the risk of PEP [41,42]. PEP rates have also been linked to pancreatic duct cannulation and/or injection and pancreatic sphincterotomy, two other procedure-related parameters (including minor papillotomy). Pancreatic duct stenting appears to lower the risk of pancreatitis, which is increased by endoscopic papillectomy [3,43].

SBC should be attempted at referral facilities by endoscopists who are familiar with the complex post-surgical anatomy seen in patients who have undergone Roux-en-Y or Billroth II gastrectomy procedures. Patients who undergo these procedures often have papilla retrograde from the gastrojejunostomy site in a section of the duodenum. Push or balloon enteroscopy retrograde from the jejunum to the duodenum is frequently required because of this additional distance in order to reach the papilla [44]. In a meta-analysis of 43 studies, success rates for biliary cannulation using spiral enteroscopy and single balloon techniques were 83% and 95%, respectively, while adverse events ranged from 0% to 3%. The studies examined single balloon, double-balloon, spiral enteroscopy, and short scope double-balloon techniques [45].

Other debated risks for PEP were related to the expertise of the operator, such as prior experience, case volume, and trainee participation; however, an accurate evaluation was proven challenging because of confounding factors including the complexity of ERCPs at high-volume facilities versus low-volume centers [46]. Only one study showed an independent rise in PEP risk with trainee involvement; however, the results were not confirmed by other authors [25,26,47]. In our institution, all of the endoscopists performing ERCP had ample expertise and a high case volume.

Some data suggest that ERCP performed in the setting of asymptomatic CBD stones is associated with a higher risk of PEP [48]. However, practice guidelines recommend ERCP to be performed regardless of symptoms, as leaving stones in place subjects the patient to a risk of often severe complications [49]. In our emergency hospital, all cases were symptomatic. Thus, a separate analysis of asymptomatic patients was not possible.

An alternative to performing ERCP and, thus, avoiding the complications associated with this procedure would be performing both laparoscopic cholecystectomy and CBD exploration in a single procedure. Although, currently, the two-stage procedure including ERCP and stone extraction followed by laparoscopic cholecystectomy is the preferred approach to CBD stones [49], recent data suggests that single-stage laparoscopic CBD exploration and cholecystectomy could represent a potentially safer and cost-efficient alternative [50]. However, ERCP presents some advantages, such as being minimally invasive, addressing small stones, being located distally in the CBD, and replacing the need for surgery in the case of residual CBD stones. In our institution, the preferred approach in the case of CBD stones is to perform ERCP followed by laparoscopic cholecystectomy during the same hospitalization. 

Our study had some limitations that were mostly related to the information available in our database. First, there was no information regarding the general cannulation time; second, the analysis did not include the time intervals of each cannulation techniques used. Moreover, information concerning late complications, such as late bleeding, was not available and was not included. 

## 5. Conclusions

PEP has been associated with the use of PS and TPBS in our institution. The highest rate of PEP was found in patients that required both of the procedures. The rate of PEP should be analyzed in each center that provides ERCP and patients should be informed of the local rate of complications prior to the procedure.

## Figures and Tables

**Table 1:** General characteristics of the patients.

Characteristic	Value
Age, median (IQR)	68 (57–76)
Sex, women/men, n (%)	119/114 (51.1/48.9)
BMI, median (IQR)	
Periampullary diverticula, n (%)	27 (11.5)
Major papilla morphology	
Type 1	151 (64.8)
Type 2	31 (13.3)
Type 3	34 (14.6)
Type 4	17 (7.3)
Indication, n (%)	
Benign conditions	
CBD stones	150 (64,4)
Biliary stricture	21 (9)
Postoperative bile duct injury	4 (1.7)
Cholangitis of unknown etiology	5 (2.1)
Malignant conditions	
Pancreatic neoplasm	23 (9.9)
Cholangiocarcinoma	22 (9.4)
Ampullary neoplasm	5 (2.1)
Malignant biliary obstruction secondary to metastatic cancer	3 (1.3)

IQR: interquartile range, BMI: body mass index, CBD: common bile duct.

**Table 2:** ERCP procedural characteristics.

Characteristic	Value
Difficult cannulation, n (%)	70 (30)
Cannulation success, n (%)	215 (92)
Cannulation technique, n (%)	
Standard wire-guided cannulation	189 (81.1)
PS	15 (6.4)
TPBS	24 (10.3)
TPBS and PS	4 (1.7)
Erlangen precut papillotomy	1 (0.4)
Periampullary diverticula, n (%)	27 (11.5)
Complications, n (%)	
PEP	23 (9.9)
Hyperlipasemia	54 (23.2)
Bleeding	7 (3)
Periampullary perforation	1 (0.4)

PS: precut sphincterotomy, TPBS: transpancreatic biliary sphincterotomy.

**Table 3:** Analysis of patient-related risk factors for PEP.

Risk Factor	No PEP 210 Cases	PEP 23 Cases	*p*
Age < 60 years old, n (%)	58 (27.6)	9 (39.1)	0.247
Female gender, n (%)	105 (50)	14 (60.9)	0.322
BMI > 24	36 (17.1)	8 (34.8)	0.044
Major papilla morphology, n (%)			
Type 1	140 (66.7)	11 (47.8)	0.072
Type 2	25 (11.9)	6 (26.1)	0.065
Type 3	30 (14.3)	4 (17.4)	0.755
Type 4	15 (7.1)	2 (8.7)	0.678
Periampullary diverticula, n (%)	26 (12.4)	1 (4.3)	0.489

PEP: Post-endoscopic retrograde cholangiopancreatography pancreatitis, BMI: Body mass index.

**Table 4:** Analysis of the relation between the types of canulation and the risk of post-ERCP complications.

Complication	Standard Wire-guided Cannulation 189 Cases n (%)	PS 15 Cases n (%)	TPBS 23 Cases n (%)	Erlangen Precut Papillotomy 1 Case n (%)	PS and TPBS 4 Cases n (%)
PEP 23 cases	14 (7.4)	3 (20)	5 (20.8)	0	1 (25)
Bleeding 7 cases	4 (2.1)	1 (6.6)	1 (4.3)	0	1 (25)
Perforation	0	0	0	0	1 (25)

PEP: Post-endoscopic retrograde cholangiopancreatography pancreatitis, PS: precut sphincterotomy, TPBS: transpancreatic biliary sphincterotomy.

## Data Availability

Data is available on demand.
